# Combination Therapy with Olmesartan and Amlodipine in the Treatment of Hypertension

**DOI:** 10.3390/ph2030125

**Published:** 2009-12-01

**Authors:** Menco G. Niemeijer, Ton J. Cleophas

**Affiliations:** 1 Academic Medical Centre, Amsterdam, The Netherlands; Email: m.g.niemeyer@gmail.com (M.G.N.); 2 Albert Schweitzer Hospital, Dordrecht, The Netherlands

**Keywords:** olmesartan, amlodipine, combination therapy, hypertension

## Abstract

*Background:* Combination therapy with antihypertensive agents utilises different mechanisms of action and may be responsible for a more effective decrease in blood pressure. *Objective:* To review the recently published trials on efficacy and safety of the combination therapy with olmesartan and amlodipine. * Results:* The double-blind American COACH (Combination of Olmesartan Medoxomil and Amlopdine Besylate in Controlling High Blood Pressure) study (2008) showed in 1,940 patients that after eight weeks of treatment the BP goals were most frequently achieved in the ‘combination therapy group’, with 56.3% (54.1–58.5%) and 54.0% (51.8–56.2%) of patients reaching adequate blood pressure of <140/90 mmHg with olmesartan/amlodipine 20/10 and 40/10 respectively. Combination therapy was generally well tolerated. The most common side effect was oedema [olmesartan 20 mg 9.9% (8.6–11.3%), amlodipine 10 mg 36.8% (34.7–39.0%), placebo 12.3% (10.9–13.8%)]. The frequency of oedema was lower in the groups combining amlodipine 10 mg with olmesartan 10 mg (26.5%, 24.5–28.5%), 20 mg (25.6%, 23.7–27.6%) or 40 mg (23.5%, 21.6–25.4%). In 2009 three double-blind controlled European studies including 500–1,000 patients each and performed independently of one another have confirmed the above study, and have demonstrated similar efficacy-safety effects from the combination of olmesartan medoxomil with amlodipine, particularly for patients not achieving adequate blood pressure control with olmesartan monotherapy. *Conclusions:* Combinations of olmesartan and amlodipine were significantly more effective at reducing blood pressure and realising guideline blood pressure goals in patients with mild to severe hypertension than monotherapy (with a placebo component). Combination therapy is well tolerated and is associated with a lower incidence of side effects, such as oedema, compared to monotherapy with high amlodipine dosages (10 mg).

## 1. Introduction

**Figure 1:**
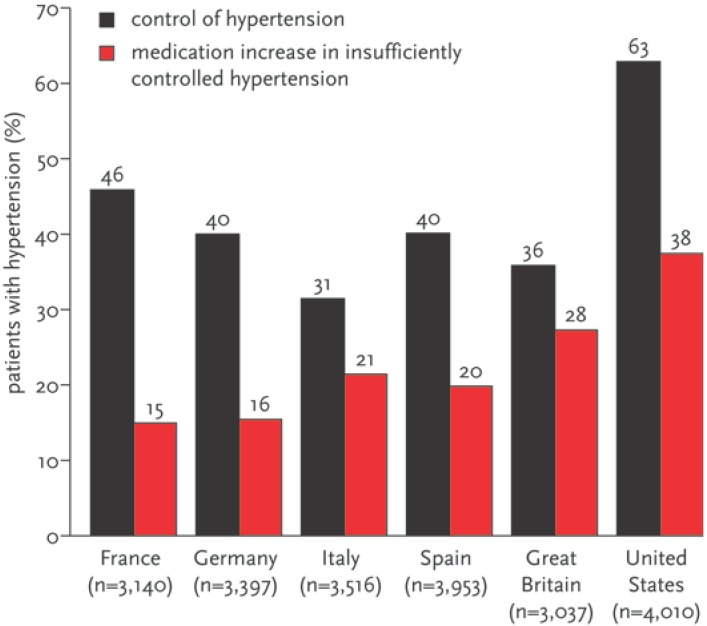
International differences in control of hypertension (systolic blood pressure <140 mmHg and diastolic blood pressure <90 mmHg) and medication increase in patients with insufficiently controlled hypertension [2] (confidence intervals were not given in the report but were small given the large samples, e.g., 44.2–47.7% and 13.8–16.2% for France).

According to the most recent data from the National Health and Nutrition Examination Survey, the prevalence of (age adjusted) hypertension in the American population, estimated from 14,653 individuals, is 29.3% (28.6–30.0%) (throughout the article 95% confidence intervals of point estimates are given) and is increasing [1]. This data corresponds to findings from studies undertaken in Western Europe [2]. Various studies have demonstrated that hypertension is clearly underdiagnosed and undertreated in both Europe and the United States (Figure 1) [2]. 

Classification of blood pressure in adults is determined in the ‘JNC 7 report’ (Table 1) [3]. The following points are important in the treatment of hypertension:

— promoting a healthy lifestyle and identifying cardiovascular risk factors and concomitant diseases that could influence the prognosis and be significant in the treatment;
— identifying demonstrable causes of high blood pressure;
— evaluating the possible presence of organ damage and cardiovascular diseases.

**Table 1:** Blood pressure classification in adults [3].

Blood pressure classification	Systolic	Diastolic
normal	<120 mmHg	and <80 mmHg
prehypertension	120–139 mmHg	or 80–89 mmHg
stage 1 hypertension	140–159 mmHg	or 90–99 mmHg
stage 2 hypertension	≥160 mmHg	or ≥ 100 mmHg

**Figure 2:**
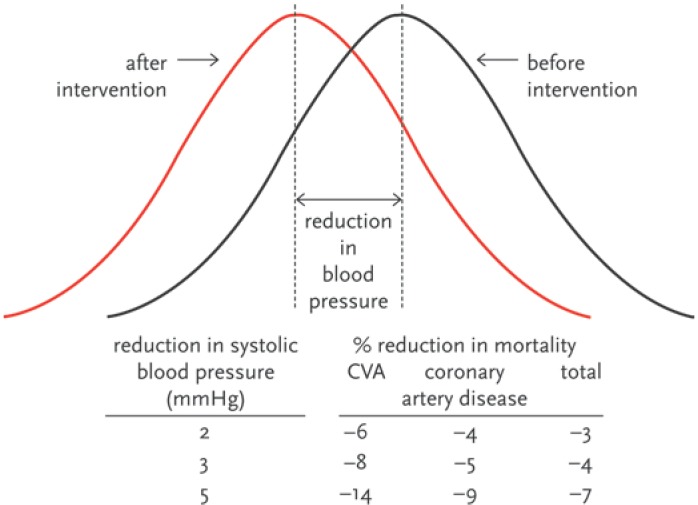
Systolic blood pressure distribution (the confidence interval were not reported, but were small given the sample size of n = 1,412, e.g., for the CVA patients respectively 4.8–7.4, 6.7–9.5, and 12.2–15.8%) [3].

A meta-analysis of 61 prospective, observational studies of over one million patients in total showed that there was a 7% (6.95–7.05%) risk reduction in cardiovascular mortality for every 2 mmHg decrease in systolic blood pressure (SBP) and even a 10% (9.94–10.06%) reduction in the risk of death from cerebro-vascular accidents (Figure 2) [3,4]. It is estimated that 370,000 coronary events in men and 150,000 coronary events in women could be prevented if blood pressure could be treated according to the blood pressure goals in the guidelines [5]. 

One of the most significant reasons for poor control is insufficient medical treatment to intensify antihypertensive therapy in patients unable to attain the blood pressure goals [6]. Extra attention to the promotion of compliance seems to be very important in reaching acceptable blood pressure. Medical guidance (from doctors or nurse practitioners) plays an essential role in this process, where the educational role in promoting compliance seems to be of particular importance. Psychological factors, such as lack of guilt, regret or shame, are strong determinants of lack of compliance [7]. 

Various extensive studies have demonstrated that it is certainly possible to achieve blood pressure goals in patients with hypertension. However, the great majority will eventually require two or more antihypertensive agents to achieve this [8]. Both the American and the European guidelines recommend starting with two antihypertensive agents in patients with an elevated cardiovascular risk or with a blood pressure exceeding 20/10 mmHg above the target value [3]. The ‘JNC 7 report’ advises the use of combinations of antihypertensive agents as initial therapy, to be administered, if indicated, in patients with stage 2 hypertension (SBP ≥ 160 mmHg or diastolic blood pressure (DBP) ≥ 100 mmHg), in particular to reach target values more rapidly, but also to avoid multiple medication ‘titration’ steps and multiple patient visits. 

## 2. The Combination with Olmesartan and Amlodipine (the COACH study)

Combination therapy with antihypertensive agents utilises different mechanisms of action and may be responsible for a more effective decrease in blood pressure [3]. One of the medicinal products in the combination could also possibly counteract the side effects of the other. For example, calcium antagonists are powerful, intrinsically natriuraemic, vasodilators resulting in a negative sodium balance and the stimulation of the renin-angiotensin-aldosterone system (RAAS), while angiotensin receptor blockers (ARBs) and angiotensin-converting enzyme (ACE) inhibitors, in contrast, block the RAAS, and, if administered in combination with calcium antagonists, in fact enhance their antihypertensive effects. Peripheral oedema, one of the side effects of dihydropyridine calcium antagonists, probably results from preferential arteriolar vasodilation and an increase in the pressure gradient between the arterial and venous capillaries, causing an exudate of interstitial fluid [9]. This effect can be improved through concurrent administration of ARBs or ACE inhibitors, which reduce the precapillary resistance, normalise the intracapillary pressure and reduce fluid exudate [3]. In addition, suppression of the RAAS has been demonstrated to protect against target-organ damage and reduce the risk of cardiovascular disease [10].

The treatment of hypertension increasingly involves a combination of an ARB and a dihydropyridine calcium antagonist, and, in the past few years, fixed drug combinations have been used in a dose that is below the dose normally prescribed when each drug is used on its own [11]. The combination of olmesartan and amlodipine is indicated in the treatment of hypertension when initial therapy to achieve the blood pressure goals is not effective. Combination therapy has been extensively investigated in factorial design studies. These compare all monotherapy doses with all possible combinations of these dosages. This enables a good comparison to be made between the effectiveness of the combination and that of the individual monotherapy, with the latter being compared with a placebo for internal validation [12].

The purpose of the COACH study (Combination of Olmesartan Medoxomil and Amlodipine Besylate in Controlling High Blood Pressure) was to investigate the effectiveness and the safety profile of combinations of olmesartan and amlodipine (Sevikar^®^) against monotherapy in patients with mild to severe hypertension [13]. The study had a multicentre, randomised, double-blind, placebo-controlled, factorial design. 

**Figure 3:**
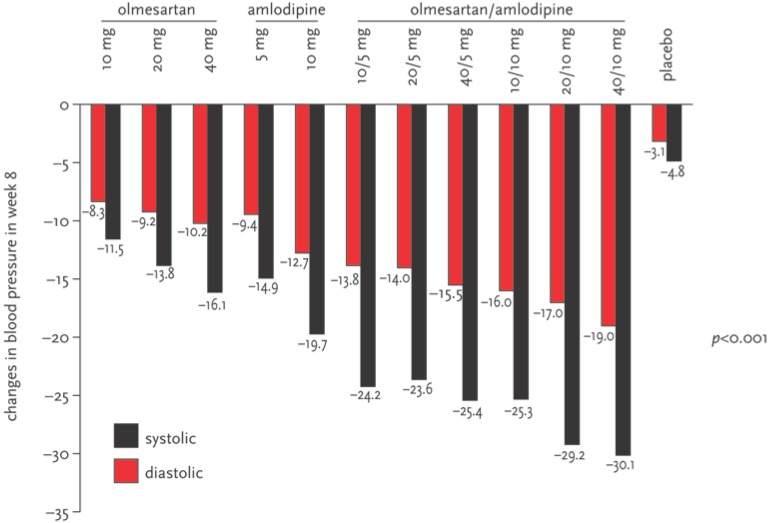
Effect of incremental dosages on diastolic and systolic blood pressure to week eight in 1,940 intention-to-treat patients [13]. Incremental dosages produced dose-dependent decreases of blood pressure. However, the combination therapies produced better decreases than did the monotherapies (p<0.001 for all equipotent dose comparisons).

54.3% (52.1–56.5%) of the 1,940 randomised patients were male. The average age was 54 years and 19.8% were over 65 years of age. The average blood pressure at the start of the study was 164/102 mmHg, and 79.3% of patients had stage 2 hypertension. The combination therapy with olmesartan/amlodipine produced decreases in diastolic blood pressure (from –14.0 mmHg with olmesartan/amlodipine 20/5 mg to –19.0 mmHg with olmesartan/amlodipine 40/10 mg) and in systolic blood pressure (from –23.6% mmHg with olmesartan/amlodipine 20/5 mg to –30.1 mmHg with olmesartan/amlodipine 40/10 mg; see Figure 3) [13]. Incremental dosages were associated with dose-dependent decreases of blood pressure. However, the combination therapies produced better decreases than did the monotherapies (p < 0.001 for all equipotent dose comparisons). 

The BP goals were most frequently achieved in the ‘combination therapy group’, with 56.3% (54.1–58.5%) and 54.0% (51.8–56.2%) of patients reaching adequate blood pressure of <140/90 mmHg with olmesartan/amlodipine 20/10 and 40/10 respectively. Combination therapy was generally well tolerated. The most common side effect was oedema (olmesartan 20 mg 9.9% (8.6–11.3%), amlodipine 10 mg 36.8% (34.7–39.0%), placebo 12.3% (10.9–13.8%)). The frequency of oedema was lower in the groups combining amlodipine 10 mg with olmesartan 10 mg (26.5%, 24.5–28.5%), 20 mg (25.6%, 23.7–27.6%) or 40 mg (23.5%, 21.6–25.4%).

## 3. Discussion

These findings of dose-dependent effectiveness with a combination of olmesartan and amlodipine are consistent with the results both from comparable studies of other fixed-dose combinations and from other studies specifically investigating a combination with a different ARB: valsartan with amlodipine [13].

There are a number of fixed-dose antihypertensive combinations, such as ARBs combined with a diuretic agent (hydrochlorothiazide) and ACE inhibitors combined with a diuretic or a calcium antagonist. The treatment that underlies these combinations is consistent with the JNC 7 recommendation, which stipulates that combinations should comprise of antihypertensive constituents that work through different mechanisms of action in order to achieve maximum therapeutic effect [3]. Both the European and the American guidelines for the treatment of hypertension indicate the importance of combination therapy in achieving the blood pressure goals more rapidly [3,14]. These guidelines recommend combination therapy, if indicated, as initial therapy when a reduction exceeding 20/10 mmHg is required to attain the blood pressure goals. However, the combination of olmesartan and amlodipine has not yet been authorised for initial therapy of hypertension. 

Fixed-dose combinations could be regarded as more patient friendly than taking two medicinal products separately. A recent meta-analysis demonstrated that compliance with fixed-dose antihypertensive combinations was significantly better than compliance with two separate medicinal products [15]. 

When a higher dose of amlodipine was combined with increasing dosages of olmesartan, the lower incidence of oedema in the COACH study was consistent with other studies, where the use of a RAAS blocker combined with amlodipine reduced the probability of amlodipine-induced oedema [13,15,16,17]. Another prominent finding of the COACH study is that it is one of the very few trials of antihypertensive combinations that *actively* investigates the development and the severity of oedema [3]. Consequently, the incidence of oedema is higher than in other studies, which only passively recorded oedema. 

One study of valsartan and amlodipine, which followed a passive recording system (side effects were ‘voluntarily’ reported by patients through asking general questions, or were diagnosed during physical examination), showed that 8.7% (2.1–26.5%) of patients developed oedema while using amlodipine monotherapy and 3.0% (0–17.2%) while using placebo [17], compared to between 13.0% (11.5–14.5%) and 36.8% (34.7–38.9%) while using amlodipine monotherapy and 12.3% (10.9–14.9%) while using placebo in the COACH study [13]. In contrast, these findings were in line with those from studies of amlodipine monotherapy that included a questionnaire with very specific questions regarding oedema. Leonetti *et al*. reported 19% (16.3–21.6%) peripheral oedema while using amlodipine 5 or 10 mg in a study with a specific questionnaire actively asking after these symptoms [18]. 

Another study (the CASTLE study), which followed a proactive monitoring system, even showed that 22.1% (16.8–27.0%) of patients who received amlodipine 5 or 10 mg had oedema [19]. In the VALUE study (also with an active monitoring system), 32.9% of patients using amlodipine 5 or 10 mg with or without hydrochlorothiazide had oedema [20]. Peripheral oedema while using ARBs in these studies developed in 8.9% (8.3–9.5%) of patients using candesartan and in 14.9% (14.2–15.6%) of patients using valsartan, which was comparable with the incidence of olmesartan in the COACH study [19,20]. 

In the last year three double blind controlled European studies including 500–1,000 patients each and performed independently of one another have confirmed the above studies, and have demonstrated similar efficacy-safety effects from the combination of olmesartan medoxomil with amlodipine, particularly for patients not achieving adequate blood pressure control with olmesartan monotherapy [21,22,23]. We should add that, since hydrochlorothiazide can also be combined with olmesartan, it may worthwhile to consider here the results of the recently published ACCOMPLISH trial performed in noless than 11,506 patients with a mean follow-up of 36 months [24]. 

## 4. Conclusions

In conclusion, combinations of olmesartan and amlodipine were significantly more effective at reducing blood pressure and realising guideline blood pressure goals in patients with mild to severe hypertension than monotherapy (with a placebo component). Combination therapy is well tolerated and is associated with a lower incidence of side effects, such as oedema, compared to monotherapy with high amlodipine dosages (10 mg). 
